# Prevalence Estimation and Genetic Characterization of Porcine Parainfluenza Virus 1 (PPIV-1) in Hungary and the First Report of the Virus in Slovakia

**DOI:** 10.1155/2024/5534854

**Published:** 2024-01-03

**Authors:** Barbara Igriczi, Lilla Dénes, Anna Czétényi, Tamás Révész, Zoltán Somogyi, Gyula Balka

**Affiliations:** ^1^Department of Pathology, University of Veterinary Medicine, István Str 2., Budapest, 1078, Hungary; ^2^National Laboratory of Infectious Animal Diseases, Antimicrobial Resistance, Veterinary Public Health and Food Chain Safety, University of Veterinary Medicine, István Str 2., Budapest, 1078, Hungary; ^3^CEVA-Phylaxia (Ceva Sante Animale), Szállás u 2, 1107, Budapest, Hungary; ^4^Department of Pharmacology and Toxicology, University of Veterinary Medicine, Budapest, István Str 2., 1078, Hungary

## Abstract

In the last few decades, many new paramyxoviruses have been discovered, causing diverse, mostly respiratory diseases in animals and humans. The porcine parainfluenza virus 1 (PPIV-1, species *Porcine respirovirus 1*), which has been reported in many countries worldwide, was found in both healthy and clinically ill pigs showing respiratory signs. Here, we report the expected prevalence and genetic diversity of PPIV-1 in Hungarian pig herds and the detection in one Slovakian pig farm, which is the first report of evidence for the presence of the virus in the country. To estimate the prevalence in Hungary 211 oral fluid samples were collected from 23 large-scale swine herds in a systematic way and tested by real-time quantitative RT-PCR. The presence of the virus was detected in 10 of the 23 Hungarian farms (43%) included in our study. One hundred eighty-one nasal swab samples were collected cross-sectionally from three Hungarian and one Slovakian PPIV-1-positive herd and PPIV-1 was most prevalent in 6-week-old pigs on farms located in Hungary and in the 2-week-old pigs on the Slovakian farm. Phylogenetic analysis of three Hungarian and two Slovakian PPIV-1 F-gene sequences showed high-nucleotide identity (>93%) and all belonged to Clade I, together with the other European strains.

## 1. Introduction

Porcine parainfluenza virus 1 (PPIV-1) species *Porcine respirovirus 1* (PRV-1) is an enveloped RNA virus belonging to the *Respirovirus* genus, *Orthoparamyxovirinae* subfamily within the *Paramyxoviridae* family. Paramixoviruses are known to affect a wide range of species, including pigs, poultry, cattle, companion animals, and also humans. Members of this family have been associated with the respiratory infections with a possible zoonotic potential [1].

PPIV-1 was first identified in nasopharyngeal—and rectal swab samples collected from slaughtered pigs between 2008 and 2012 in Hong Kong, China [2]. Full genome sequencing and phylogenetic analyses showed that this novel paramyxovirus is most closely related to human parainfluenza virus 1 (HPIV-1) and Sendai virus (SeV) within the *Respirovirus* genus. In the last years, the virus was detected in the USA [3–5], Chile [6], Hungary [7], Germany, the Netherlands [8], Poland [9], and in the Republic of Korea [10].

The nonsegmented, negative sense, single-stranded RNA genome of PPIV-1 is around 15 kilobases in length and encodes six major proteins (3-N-P-M-F-HN-L-5′): nucleocapsid (N), phosphoprotein (P), matrix (M), fusion (F), hemagglutinin–neuraminidase (HN), and large proteins (L), respectively. The HN and F proteins are major surface glycoproteins that are responsible for interaction with the host cell. The HN protein plays a role in the binding and entry of the virus, whereas the F protein mediates the fusion of the viral envelope and the cell membrane. Although limited number of full genome sequences are publicly available, HN and F genes with high-genetic variability can be used for genetical and epidemiological studies on PPIV-1 [5, 8]. Phylogenetic analysis of the complete nucleotide sequence of the F protein revealed that PPIV-1 divides into two distinct clades [11]. Lineage 1 contains sequences from Europe and Hong Kong, and while lineage 2 contains sequences originating from the American and Asian continents, suggesting divergent evolution of European and American strains [11].

The exact pathogenic role of the virus is currently unknown, but PPIV-1 infection has been associated with respiratory diseases. The virus was often detected in pigs displaying respiratory symptoms, such as coughing, sneezing, and nasal discharge [3, 6, 9, 10]. Histopathological and in situ hybridization studies showed that the virus replicates in the nasal and tracheal respiratory epithelial cells [3]. In a recent study, an experimental challenge performed on 4-week-old conventional (CON) and 6-week-old cesarean derived/colostrum deprived (CD/CD) pigs resulted in high-viral quantities in all respiratory samples with nasal viral shedding and replication in the upper and lower respiratory tract [12]. Despite the high levels of PPIV-1 replication, only mild clinical respiratory symptoms were observed. Several other studies showed that the viral genome was also present in asymptomatic pigs [2, 5, 7, 8]. Palinski et al. [3] reported that 11, randomly selected, naturally infected pigs showed no signs of coughing, sneezing, nasal discharge, or lethargy during a 2-week observation period.

These findings suggest that PPIV-1 infection itself may not play a primary role in respiratory diseases, but coinfections with other pathogens, such as porcine reproductive and respiratory syndrome virus (PRRSV) and/or influenza A virus (IAV) may contribute to porcine respiratory disease complex (PRDC), increasing the severity of the disease outcome [9, 13].

PPIV-1 in Europe was first described by our research group in nasal swab samples originating from Hungarian farrow-to-finish farms [7]. Among the 22 herds examined in our study, only one (4.5%) was positive for PPIV-1. However, the prevalence of this virus seems relatively high in other European countries, as its presence was reported on 42.3% of German and Dutch farms [8], and 76.7% of Polish farms [9]. Also, in Chile Agüero et al. [6] found the virus in all six farms tested, suggesting that PPIV-1 is widely disseminated in the country. In the present study, our aims were to screen large-scale pig herds in a systematic way to estimate the prevalence of PPIV-1 and to investigate the genetic diversity of the Hungarian strains.

## 2. Materials and Methods

### 2.1. Sample Collection

**Figure 1:**
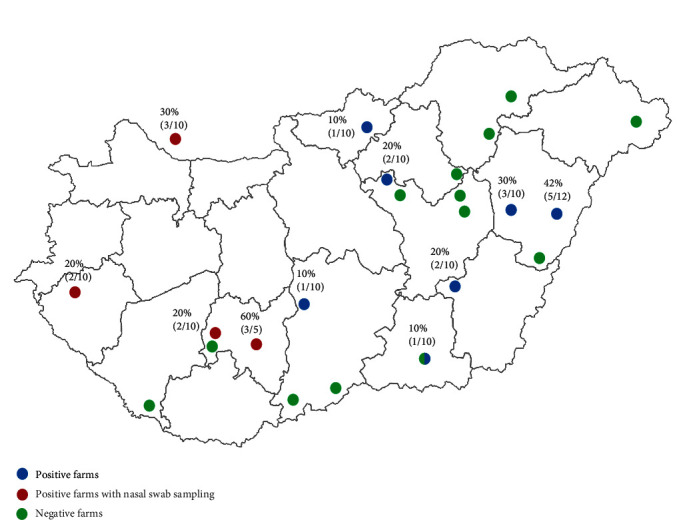
Map of Hungary highlighting the geographic location of the sampled farms (red/green/blue dots). The percentages of the PPIV-1-positive oral fluid samples on each farm are indicated on the map.

All samples were gathered from 23 large-scale Hungarian pig herds between 2020 and 2022, as a part of an active surveillance sampling program (ethical permission number: PE/EA/544-5/2018; Figure 1). We also received samples from a Slovakian pig farm located close to the Hungarian border for diagnostic purposes. The farms varied in basic production parameters, sow herd size and genetics but were all farrow-to-finish. The participation in the sampling campaign was voluntary of all farms regardless of their health status, and no significant clinical disease was reported in the herds during the sampling period. On most farms, according to the protocol, 100 blood samples were drawn from animals of different age groups, 5 pen-based oral fluid samples were collected from weaned pigs (8–12 weeks of age; WOA), and 5 samples from fatteners (18–20 WOA) [14]. More details of the sampling protocol can be found in the study of Igriczi et al. [15]. Upon the request of the farm owners and/or farm veterinarians—after recognizing the presence of the virus in their herds—additional cross-sectional samplings were also carried out in three cases: nasal swabs were collected from 2-, 4-, 6-, and 8-week-old animals. Nasal swab samples from the Slovakian farm were originally sent in for swine influenza virus (SIV) screening, because the animals in the nursery unit showed clinical signs of respiratory disease. The samples from this farm were collected cross-sectionally from 2-, 6- and 8-week-old animals. Altogether 221 oral fluid samples and 181 nasal swabs were collected and were all stored at −80°C until further use.

### 2.2. Sample Processing and RNA Extraction

The oral fluid samples were tested individually and centrifuged at 300 × g for 5 min before extraction. Nasal swabs were vortexed in PBS and equal volumes (100 *µ*l) of samples from the same age groups were pooled by 4 or 5. The samples of PPIV-1-positive pools were processed later individually. The RNA was extracted from 200 *µ*l of pooled or individual nasal swab and oral fluid samples by QIAcube automatic nucleic acid extractor (Qiagen, Hilden, Germany) using the QIAmp cador Pathogen Mini Kit (Qiagen) according to the manufacturer's protocol. The RNA samples were stored at −80°C until further analysis.

### 2.3. RT-qPCR Detection of PPIV-1

Real-time quantitative reverse transcription PCR (RT-qPCR) was performed to detect the L gene of PPIV-1 in the samples with a subsequent melting point analysis. The RT-qPCR assays were run on Rotor-Gene Q instrument (Qiagen) using QuantiNova SYBR Green RT-PCR Kit (Qiagen) and specific primers (forward primer: 5′-TACAATATATGTGGGTGATCCTTACT-3′ and reverse primer: 5′-GCCTGAATCTTCATGATCTTCTAAA-3′) previously published by Lau et al. [2], with the following temperature profile: 50°C for 10 min, 95°C 30 min followed by 40 cycles of 95°C for 5 s, and 60°C for 30 s. Samples with Ct (cycle threshold) values higher than 37 were considered negative.

The RT-qPCR results were statistically analyzed using GraphPad Prism 8 for Windows. The Ct values of the different age groups and sample types were compared using the Mann–Whitney test.

### 2.4. PPIV-1 Fusion (F) Protein Gene Sequencing and Phylogenetic Analysis

Nasal swab samples from PPIV-1 positive pools were extracted and tested individually. Sequencing of the F gene was attempted on samples with the lowest Ct values. The reactions were performed in a Genesy 96T gradient PCR machine (Tianlong, China) using One-Step RT-PCR Kit (Qiagen) and specific internal and outer primer pairs (F-Rev: 5′-TCGTGCACCCTAAGTTTTCTTTA-3′ and F-For int 1 : 5′-GAGAGAAGAGCTTAACATTACAGGC-3′; F-For: 5′-ACTTAGGGTACAAGTTATCCAAAAAA-3′ and F-Rev int 1 : 5′-TCATAAATATCTGTYTTCCCGAGATT-3′) described by Park et al. [5]. The PCR reactions were run under the following temperature profile: 48°C for 20 min; 94°C for 3 min followed by 40 cycles of 94°C, 30 s; 55°C, 50 s; 68°C, 2 min; and one last step at 68°C for 7 min. After agarose gel electrophoresis, amplicons with the expected size were manually cut and purified from agarose gel using QIAquick Gel Extraction Kit (Qiagen). BigDye^TM^ Terminator v3.1 Cycle Sequencing Kit (Thermo Fisher Scientific, Ljubljana, Slovenia) was used for the Sanger sequencing reaction. For purification and concentration of high-quality DNA from PCR reactions DNA Clean & Concentrator®-5 (Zymo Research, Irvine, CA, USA) was used. Capillary electrophoresis was performed by a commercial provider (BIOMI Kft., Gödöllő, Hungary).

Visual inspection and trimming of all chromatograms were carried out using Chromas 2.6.6 software (Technelysium Pty Ltd, South Brisbane, Australia). The forward and reverse sequences were assembled and aligned against all available PPIV-1 F gene sequences downloaded from the GenBank, using the E-INS-I method of the MAFFT version 7 online software [16]. Maximum-likelihood phylogenetic trees were constructed using MEGAX [17] performing bootstrap analysis with 1,000 replicates.

## 3. Results

### 3.1. Detection Rates of PPIV-1 in Different Pig Farms

**Figure 2:**
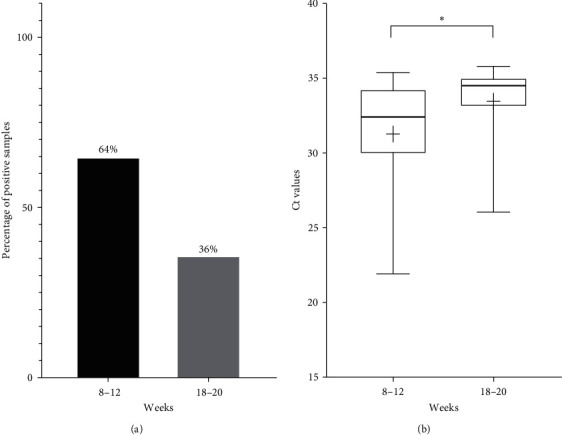
(a) Percentages of PPIV-1 positive oral fluid samples of weaned pigs (8–12 WOA) and fatteners (18–20 WOA). (b) Boxplots displaying the distribution of Ct values in PPIV-1-positive oral fluid samples of weaned pigs (8–12 WOA) and fatteners (18–20 WOA). The whiskers indicate the lowest and highest values, while the “+” signs show the average. The horizontal lines of the box represent the first quartile, the median and the third quartile. The asterisk above the boxes indicates the statistically significant difference ( ^*∗*^*P* < 0.05).

PPIV-1 was detected in 11 of the 24 farms (46%) involved in our study (Figure 1) and the estimated Hungarian prevalence was 43% (10/23). Altogether, 25 of the 221 oral fluid samples (11%) were positive for PPIV-1 and the percentage of the positive samples varied between 10% and 60% in the positive herds. Sixty-four percent (16/25) of the PPIV-1-positive samples belonged to the weaned pigs and 36% (9/25) belonged to the fatteners (Figure 2). Considering the viral quantities, the Ct values of the positive oral fluid samples ranged from 21.92 to 35.39 for weaned pigs and from 26.05 to 35.79 for fatteners. The Ct values of the PPIV-1 positive 8–12-week-old pigs' samples were significantly lower (mean Ct value: 31.27 ± 3.97) than what was detected in the samples of the 18–20-week-old pigs (mean Ct value: 33.46 ± 2.94; Figure 2).

**Figure 3:**
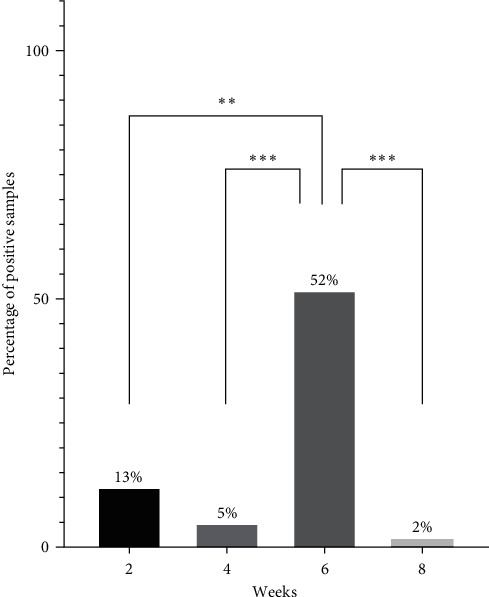
Percentages of PPIV-1-positive nasal swabs of different age groups. Statistical analysis comparing the PPIV-1 prevalence across different age groups was conducted using Fisher's exact test. The asterisks above the columns represent the statistically significant difference ( ^*∗*^*P* < 0.05,  ^*∗∗*^*P* < 0.01, and  ^*∗∗∗*^*P* < 0.001).

**Figure 4:**
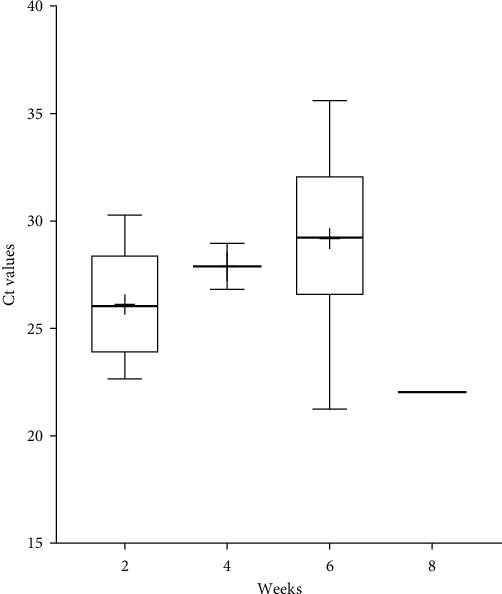
Boxplots displaying the distribution of Ct values in PPIV-1-positive nasal swabs of different age groups. The whiskers indicate the lowest and highest values, while the “+” signs show the average. The horizontal lines of the box represent the first quartile, the median and the third quartile.

**Figure 5:**
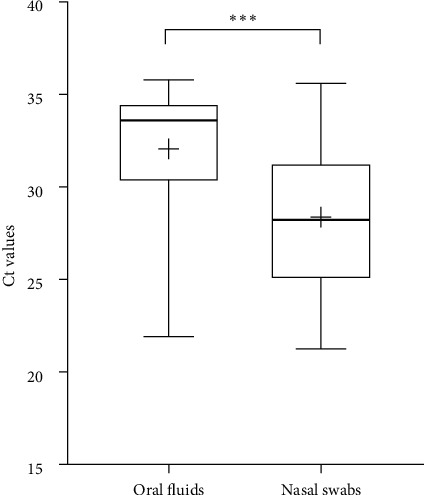
Boxplots displaying the distribution of Ct values in PPIV-1-positive oral fluid and nasal swab samples. The whiskers indicate the lowest and highest values, while the “+” signs show the average. The horizontal lines of the box represent the first quartile, the median and the third quartile. The asterisks above the boxes indicate the statistically significant difference ( ^*∗∗∗*^*P* < 0.001).

PCR positive nasal swab samples were found on all four farms where nasal swab sampling was also performed. Of the 181 nasal swab samples, 34 samples (18.8%) from four pig farms were positive for PPIV-1. Among the different age groups, the detection rate was significantly higher in the samples obtained from 6-week-old animals than in any other age groups tested, as more than half of the samples (52%) were positive (Figure 3). The Ct values of the positive nasal swabs ranged from 21.24 to 35.06, with the mean Ct value of 28.37 ± 3.84 (Figure 4). There were no significant differences between the Ct values measured in each age group. However, statistical comparison of the different sample types showed that the Ct values of the PPIV-1-positive nasal swabs were significantly lower than the oral fluids, with the mean Ct value of 32.06 ± 3.73 (Figure 5). Moreover, serum samples from the four PPIV-1-positive farms with nasal swab sampling were also tested with RT-qPCR, but all samples were negative for PPIV-1.

### 3.2. Different Circulation Patterns on Four PPIV-1 Positive Herd

**Figure 6:**
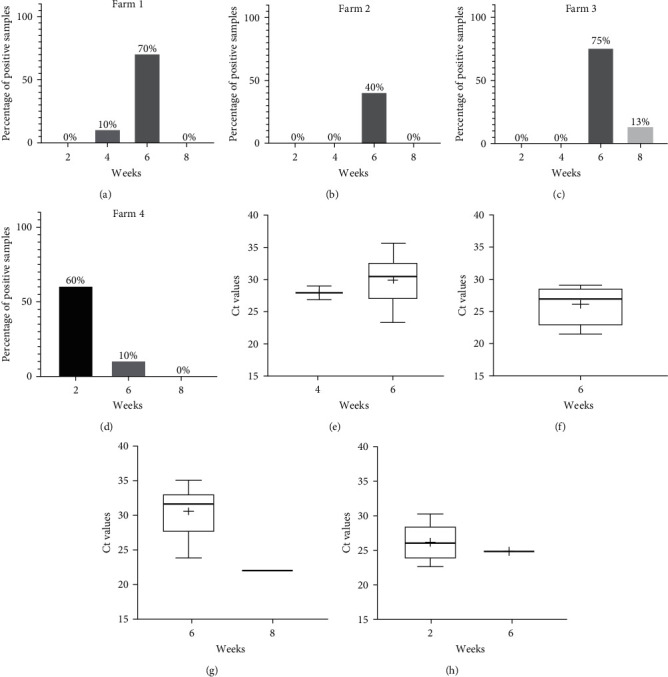
(a–d) Percentages of PPIV-1 positive nasal swabs of different age groups from Farm 1 (a), Farm 2 (b), Farm 3 (c), and Farm 4 (d). (e–h) Boxplots representing the Ct values of the PPIV-1 positive nasal swabs different age groups from Farm 1 (e), Farm 2 (f), Farm 3 (g), and Farm 4 (h). The whiskers indicate the lowest and highest values, while the “+” signs show the average. The horizontal lines of the box represent the first quartile, the median and the third quartile.

To gain information about the within-herd infection dynamics of the virus we performed cross-sectional nasal swab sampling in four PPIV-1-positive herds. We found some differences in the patterns of PPIV-1 circulation on these farms (Figure 6). On Farms 1, 2, and 3, located in Hungary, the detection rates were the highest in the 6-week-old piglets (75%, 70%, and 40%, respectively). On Farm 1, the virus already appeared in 4-week-old piglets and on Farm 3 some samples from the 8-WOA group were also positive. On the other hand, on Farm 4 located in Slovakia, the highest detection rate (60%) was observed in the youngest, 2-WOA group. The estimated prevalence on this farm seemed to decrease toward the older age groups. Pairwise statistical comparisons however showed no significant differences between the Ct values of the PPIV-1-positive nasal swabs from the different age groups. On Farm 1, there were no significant differences between the Ct values measured in 4- and 6-week-old animals. On the other three farms, pairwise statistical comparisons could not be performed as there were too few positive samples in some age groups.

### 3.3. Sequence Analysis and Phylogenetics

**Figure 7:**
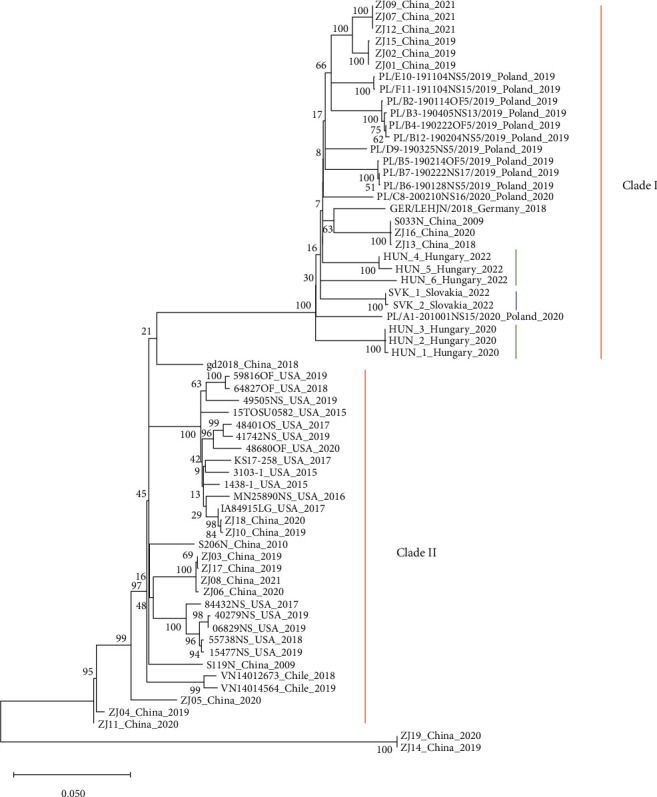
Phylogenetic analysis of the F-gene of PPIV-1 sequences from GenBank and Hungarian (marked with green) and Slovakian (marked with blue) strains. The phylogenetic tree was built using MEGAX with maximum likelihood method and conducting 1,000 bootstrap replicates for analysis. All available PPIV-1 F-gene sequences were downloaded from GenBank and aligned against the sequences detected in this study.

Nasal swabs with the lowest Ct values were selected from each PPIV-1 positive farm for partial genome sequencing. We were able to obtain the sequence of the Fusion polyprotein coding gene in five cases: one sequence from each Hungarian farm and two from the Slovakian samples (GenBank accession numbers: OQ877210–OQ877214). Comparative nucleotide sequence analysis revealed 93.53%–94.65% similarity between the F genes of Hungarian and Slovakian strains. The genotypic classification of the detected PPIV-1 sequences was based on the method proposed by Stadejek et al. [11]. Accordingly, all PPIV-1 strains can be divided into two genetic lineages which cluster into two distinct clades on the phylogenetic trees. The Hungarian and Slovakian strains detected in this study belonged to Clade I and their similarity with the previously described sequences ranged from 65.93% to 95.59% and from 66.29% to 95.18%, respectively (Figure 7).

## 4. Discussion

Members of the family, *Paramyxoviridae* are responsible for different highly infectious diseases with the tendency to spread across species. So far three members of this family, including the porcine rubulavirus (blue eye disease) [18], Menangle virus [19], and Nipah virus [20] have been associated with the clinical disease in swine. In the recent years, PPIV-1 was detected in different countries all over the world, but the pathogenicity of the virus in pigs is still unclear. PPIV-1 infection has been connected with respiratory symptoms [3, 6, 9, 10, 12], but some studies reported that the virus can also circulate in clinically healthy farms [7, 8]. In 2020, for the first time in Europe, our research group reported the presence of the virus in a Hungarian pig farm [7]. In the last 2 years, we conducted a widespread systematic sample collection from all over the country to obtain more relevant information on the PPIV-1 prevalence in Hungary. We also investigated samples that were received from a Slovakian pig farm located close to the Hungarian border for diagnostic purposes.

Our results indicate that PPIV-1 is widely spread in Hungary, as the presence of the virus was detected in 10 of the 23 Hungarian farms (43%) included in our study (Figure 1). We also detected the virus in a Slovakian farm, which represents the first scientific evidence of the presence of the virus in the country. After the initial positive oral fluid sampling, a cross-sectional nasal swab sampling was performed on three PPIV-1-positive Hungarian herds. Samples from the Slovakian farm came along with nasal swabs for SIV screening. The virus was detected in nasal swabs in all four farms, indicating ongoing PPIV-1 infection in the respiratory tract of the positive individuals.

In our previous study, the virus was detected in only 1 of the 22 Hungarian farms (4,5%) involved [7]. This low-prevalence rate might be explained with the different sampling protocols: in our first study, nasal swab samples from 3-week-old animals were collected as a part of a neurotropic astrovirus screening study [21]. In the present work, we used pen-based oral fluid samples collected from weaned pigs and fatteners. This method allowed us to screen more animals at once from different production units. Similarly to our results, the presence of PPIV-1 was confirmed in 42.3% (11/26) of the herds examined in Germany and the Netherlands [8]. Studies conducted in Chile by Agüero et al. [6] and in Poland by Woźniak et al. [9] targeted herds where respiratory clinical signs were observed at the time of sampling. The authors reported higher detection rates compared to Hungary as 100% (6/6) and 76.7% (23/30) of farms were PPIV-1-positive, respectively. The higher PPIV-1 prevalence in herds with respiratory disease suggests that natural PPIV-1 infection might be involved in the pathogenesis of respiratory diseases, although the virus alone may not be sufficient to induce clinical disease.

The results of previous studies indicate that PPIV-1 infection occurs mainly in the younger age groups [7, 9]. To gain more information on the viral circulation on pig farms, we analyzed samples obtained in a cross-sectional way on four different, previously PPIV-1-positive herds. To our knowledge this is the first comparative, within-herd infection dynamics study regarding PPIV-1. Analyzing the patterns detected on each farm we found that PPIV-1 was most prevalent in 6-week-old pigs on Farms 1, 2, and 3 (40%–75% positivity), located in Hungary. Interestingly, on these farms the virus was absent from the youngest age group (2-WOA), and only 10% of the 4-week-old animals were positive for PPIV-1 in one of the farms. These results might suggest the protective effect of maternally derived immunity, which seemed to protect the animals until weaning on these farms. Although the prevalence in the 6-WOA group was relatively high, we found only one PPIV-1-positive sample among the 8-week-old animals. Interestingly, on Farm 4, located in Slovakia, the virus was most prevalent in the 2-WOA group, which might suggest that the piglets received inadequate quantities or qualities of colostrum, so the virus could spread quickly amongst them (Figure 6). The highest viral burden in this farm was observed among the 2-week-old animals, as the mean Ct value was 26.18 ± 2.76, while in the 6-WOA group it was 29.18 ± 3.88. Pairwise statistical comparisons however showed no significant differences between the Ct values of the PPIV-1-positive nasal swabs from the different age groups (Figure 4). As these samples from the Slovakian farm were originally sent in for SIV screening, we have to mention that only one sample from the 8-WOA group was RT-qPCR positive for SIV according to the protocol published by Nagy et al. [22].

To our knowledge, there are no data published on the role of maternal immunity in the case of PPIV-1 infection, but our results indicate that weaned pigs are more susceptible to the infection and the virus circulates mostly among the 6-week-old animals which might coincide with the fading levels of the antibodies as it has been observed for other respiratory viruses [23–25]. The prevalence of infection in the different age groups is in harmony with a recently published study from Poland where 46.9% of the nasal swabs collected from the nursery units were PPIV-1-positive, and the virus was the most prevalent among the 5- and 7-week-old pigs [9]. In our previous study, where a similar cross-sectional sampling protocol was used in one PPIV-1-positive herd, the virus was most prevalent among the 4-week-old pigs (65%), but 40% of the 6-week-old pigs' samples were also positive [7].

Comparison of Ct values across two distinct diagnostic matrices revealed that PPIV-1-positive nasal swabs had significantly lower Ct values than the oral fluids (Figure 5). The higher viral loads, coupled with lower contamination risks make nasal swab samples suitable for virus diagnosis. However, for surveillance at the herd- or group-level, oral fluid samples emerge as a practical alternative due to their ease of collection and ability to represent a collective status.

Phylogenetic analysis of the PPIV-1 F-gene showed that the three Hungarian and the two Slovakian sequences obtained in this study belonged to Clade I, together with other European and some Chinese strains (Figure 7). The nucleotide identity of the Hungarian and Slovakian strains was more than 93% and both of them showed close genetic relation with a recently submitted Polish PPIV-1 sequence (95.59% and 95.18% respectively).

This study confirms that PPIV-1 is more widespread in Hungary than we assumed in our previous study, and the presence of the virus was also confirmed in Slovakia for the first time. Even though, there were no overt respiratory clinical signs reported at the time of the samplings, the viral burden of the PPIV-1-infected weaned pigs was relatively high. These results suggest that the viral infection alone may not cause clinical symptoms, but according to the previous studies, PPIV-1 can possibly be a component of PRDC [9, 10, 13]. Further studies on herds with respiratory clinical symptoms are needed in order to understand the pathogenesis and clinical relevance of PPIV-1 and to determine its possible role in the PRDC.

## Data Availability

Sequence data gathered during the study can be accessed under GenBank accession numbers: OQ877210–OQ877214. Data regarding the name and exact location of the farms involved in the study are confidential due to business secret of the owners.
